# A first-in-human phase I study of TAS0728, an oral covalent binding inhibitor of *HER2*, in patients with advanced solid tumors with *HER2* or *HER3* aberrations

**DOI:** 10.1007/s10637-021-01104-7

**Published:** 2021-03-27

**Authors:** Sarina A. Piha-Paul, Analía Azaro, Hendrik Tobias Arkenau, Do-Youn Oh, Matthew D. Galsky, Sumanta Kumar Pal, Kensuke Hamada, Yaohua He, Ikuo Yamamiya, Karim A Benhadji, Antoine Hollebecque

**Affiliations:** 1 Department of Investigational Cancer Therapeutics, Division of Cancer Medicine, The University of Texas MD Anderson Cancer Center, Houston, TX USA; 2 Department of Medical Oncology, Vall d’Hebron Institute of Oncology (VHIO), Barcelona, Spain; 3 Sarah Cannon Research Institute, University College Hospital, London, UK; 4 Department of Internal Medicine, Seoul National University Hospital, Cancer Research Institute, Seoul National University College of Medicine, Seoul, South Korea; 5 Novel Therapeutics Unit, Center of Excellence for Bladder Cancer, The Tisch Cancer Institute and Icahn School of Medicine, Mount Sinai, New York City, New York USA; 6 Department of Medical Oncology and Therapeutics Research, City of Hope National Medical Center, Duarte, California USA; 7 Taiho Oncology, Inc., Princeton, NJ USA; 8 Drug Development Department, Cancer Campus, Institut Gustave Roussy, 114, Rue Édouard-Vaillant, 94805 Villejuif, France

**Keywords:** TAS0728, Human epidermal growth factor receptor 2, Erb-B2 receptor tyrosine kinase 3, Phase I study, Neoplasms

## Abstract

TAS0728 is an oral covalent binding inhibitor of human epidermal growth factor receptor 2 *(HER2).* A first-in-human open-label, dose-escalation, phase I study (NCT03410927) was initiated to investigate the safety and dose-limiting toxicity (DLT) and to determine the maximum tolerated dose (MTD) and/or recommended phase II dose of TAS0728 in adults with advanced solid tumors with *HER2* or *HER3* overexpression, amplification or mutation. In total, 19 patients received TAS0728 at escalating doses from 50 to 200 mg BID for 21-day cycles. Following escalation of the dose to 200 mg BID, a total of two DLTs were observed, both cases of Grade 3 diarrhea (lasting >48 h and not responsive to aggressive antidiarrheal treatment). Following de-escalation of the dose to 150 mg BID, another DLT of Grade 3 diarrhea was observed in one patient. Additionally, at 150 mg BID, one patient had a fatal cardiac arrest after receiving 1 cycle (21 days) of TAS0728. The etiology of the cardiac arrest event was not clear, however causal relationship to TAS0728 could not be excluded due to the temporal association observed. Partial responses were observed in 2 of 14 patients evaluable for TAS0728 treatment response. The study was stopped due to unacceptable toxicity during the dose-escalation as the overall risk-benefit ratio no longer favored the dose level being tested, therefore the MTD was not determined. ClinicalTrials.gov registration number: https://clinicaltrials.gov/ct2/show/NCT03410927; registered on January 25, 2018.

## Background

### Introduction

Both amplification and mutation of *HER2* and *HER3* have been associated with various tumors and targeting of *HER2* has shown efficacy in treating breast and gastric/gastroesophageal cancers [1]. Although anti-HER2 antibodies, including trastuzumab, pertuzumab, and the antibody–drug conjugates, trastuzumab emtansine (T-DM1, Kadcyla®), and trastuzumab deruxtecan (Enhertu®), are approved for treating *HER2*-overexpressing breast cancers, effective therapies are needed for patients who are refractory to HER2-targeting antibodies [2].

Various covalent-binding irreversible inhibitors of HER2 exhibit robust and sustained target engagement in preclinical models [3]. However, these reported HER2-inhibitory covalent binders are not selective for HER2 and instead act as pan-ErbB tyrosine kinase inhibitors (TKIs) that block the activity of ErbB family kinases, including epidermal growth factor receptor (EGFR) [3]. Inhibition of EGFR can result in dose-limiting rashes and gastrointestinal issues, particularly diarrhea to the level of Grade 3 or 4 toxicity (as observed in the LUX-BREAST-1/−3 studies of afatinib [4, 5] and the ExteNET trial of neratinib [6] in HER2-positive breast cancer). Therefore, novel agents having greater specificity for HER2 inhibition while excluding EGFR may overcome the resiliency of the HER2/HER3 pathway in HER2-activated cancers and improve the clinical response rates versus conventional HER2 TKIs.

TAS0728 is an orally available, HER2-selective covalent inhibitor with high specificity for HER2 over wild-type EGFR and has exhibited potent inhibitory activity for both overexpressed/amplified *HER2* and mutated *HER2* in cancer cells [2]. TAS0728 has demonstrated antiproliferative activity against *HER2* overexpressed cancer cells in a dose-dependent manner in vitro and in vivo [2]. In xenograft models of tumors with acquired resistance to trastuzumab/pertuzumab or to T-DM1, HER2 kinase inhibition with TAS0728 produced significant anti-tumor effects [7]. A first-in-human phase I dose-escalation study was initiated to investigate safety and dose-limiting toxicity (DLT) and to determine the maximum tolerated dose (MTD) and/or recommended phase II dose (RP2D) of TAS0728 in patients with advanced solid tumors with *HER2* or *HER3* aberrations.

### Methods

#### Study population

Patients aged ≥18 years old with locally advanced, recurrent or metastatic, histologically confirmed advanced solid tumors with *HER2* or *HER3* overexpression, amplification or mutation who had failed all standard therapies or for whom standard therapy did not exist were eligible for inclusion. Patients had an Eastern Cooperative Oncology Group (ECOG) performance status of 0 or 1; and measurable or evaluable disease with either *HER2*-positive status (immunohistochemistry (IHC)3+ and/or fluorescence in situ hybridization (FISH)+) or a potentially actionable *HER2* or *HER3* mutation determined by local laboratory. Patients could have received ≤2 different forms of specific anti-*HER2* therapy for their cancer previously (≤4 lines of anti-*HER2* therapy for breast cancer cases).

Patients needed to have the following laboratory values: absolute neutrophil count (ANC) ≥1.5 × 10^9^/L; hemoglobin ≥8.0 g/dL; platelet count ≥75 × 10^9^/L; albumin ≥3 g/dL; serum potassium, magnesium, phosphorus, sodium, total calcium (corrected for serum albumin) or ionized calcium within institutional normal limits; aspartate transaminase/serum glutamic-oxaloacetic transaminase and alanine aminotransferase/serum glutamic-pyruvic transaminase ≤3× upper limit of normal (ULN) or ≤ 5.0x ULN if liver metastases were present; total serum bilirubin ≤1.5× ULN; serum creatinine ≤1.4x ULN or 24-h or calculated creatinine clearance (CrCl) ≥50 mL/min (for a calculated CrCl value, the eligibility was determined using the Cockcroft-Gault formula).

Patients were excluded from the study if they had a history of brain metastases or another primary malignancy; impaired cardiac function or clinically significant cardiac disease; recent treatment (within 5 half-lives of the drug or within 4 weeks of the first planned study dose) with chemotherapy, biologic therapy, targeted therapy, immunotherapy, extended-field radiotherapy, or investigational agents; or recent major surgery (within previous 4 weeks).

#### Study design

This open-label, phase I study (ClinicalTrials.gov: NCT03410927) was designed to evaluate the safety, pharmacokinetics (PK), and efficacy of TAS0728 in patients with advanced solid tumors with *HER2* or *HER3* aberrations who had progressed despite standard therapy or for which no standard therapy existed. The protocol was approved by the Independent Ethics Committee or Institutional Review Board at all participating centers and the study was conducted in accordance with the ethical principles laid out in the Declaration of Helsinki. All patients provided written informed consent prior to enrollment.

#### Drug dose and administration

Based on preclinical toxicological studies and PK analysis, the starting dose of TAS0728 was 50 mg BID. Dose escalation followed a 3 + 3 dose-escalation scheme and planned to proceed using the following dose levels BID: 50 mg, 100 mg, 200 mg, 400 mg, 600 mg and 800 mg. TAS0728 was administered during a 21-day cycle. If no DLT was observed in a cohort of 3 patients at a given dose level, the next cohort of 3 new patients was to be enrolled at the next higher dose level.

If a DLT was identified at a particular dose level, de-escalation was made to an intermediate de-escalation dose level. If 2 DLTs were observed in a dose escalation level, then the dose escalation level below that at which DLTs occurred would be expanded as the potential MTD level until 6 DLT-evaluable patients had been treated at that dose level. The MTD was to be the dose level at which 0 of 6 or 1 of 6 patients experienced a DLT, with at least 2 patients experiencing DLTs at the next higher dose level. For the determination of the MTD, DLTs that occurred during the first cycle (i.e., first 21 days) of TAS0728 treatment were considered. Patients who experienced AEs were allowed two dose reductions during the study. TAS0728 treatment could be continued until disease progression, unacceptable toxicity, withdrawal of consent, or at the discretion of the investigator.

#### Dose limiting toxicities (DLTs)

A DLT was defined as a study treatment-related adverse event (TRAE), according to the Common Terminology Criteria for Adverse Events (CTCAE, Version 5.0), with qualifying criteria for certain DLTs (e.g., Grade ≥ 3 diarrhea, was included as a DLT only if lasting >48 h and unresponsive to intensive antidiarrheal medication).

#### Safety assessments

Based on preclinical toxicity studies, toxicities of note were hematologic toxicity in the form of lymphopenia, blood chemical toxicities, such as increased serum amylase and lipase, and gastrointestinal toxicities, such as diarrhea and nausea. Patients were closely monitored for these potential toxicities during the current study. Safety assessments included recording of AEs and serious AEs (SAEs) from the time of signing the study informed consent form to 30 days after the last dose of study medication. Safety parameters including laboratory evaluations (hematology, coagulation, chemistry, and urinalysis), vital sign measurements and body weight, electrocardiogram (ECG) recordings, ECOG performance status, echocardiogram or multi-gated acquisition (MUGA), and physical examination were assessed at screening, during the study and at 30 days following the last dose. The use of concomitant medications was permitted and details for such medications were recorded.

#### Pharmacokinetics

Multiple blood samples were collected on day 1 of cycles 1 and 2 (i.e., pre-dose, post-dose at +0.5 h, +1 h, +1.5 h, +2 h, +3 h, +4 h, +6 h, +8 h, +12 h) for analysis of plasma PK after administration of single and multiple doses respectively; urine samples were collected 0–3 h prior to morning dose and 0–12 h post-morning dose on day 1 of cycle 1.

#### Antitumor activity

Objective tumor assessments were made according to the revised response evaluation criteria in solid tumors [RECIST1.1] [8] from evaluation of magnetic resonance imaging (MRI)/computed tomography (CT) scans made at screening (baseline) and after every 2 cycles during the first 6 months of treatment with TAS0728, and after every 3 cycles of treatment thereafter.

#### Statistical analysis

Continuous data were summarized with frequency, median, range, mean, standard deviation and standard error if relevant. Categorical data were presented as frequencies and percentages. 95% confidence intervals (CI) were calculated following the exact method. A *p* value <0.05 was considered statistically significant. Data were analyzed using the SAS® system software version 9.3 for Windows® (Statistical Analysis System, Cary, NC, USA). The PK data were analyzed using Phoenix WinNonlin (Version 6.4 or later, Certara L.P; Princeton New Jersey, United States). All analyses were descriptive and exploratory; no formal statistical testing was conducted.

### Results

#### Patient characteristics

**Fig. 1:**
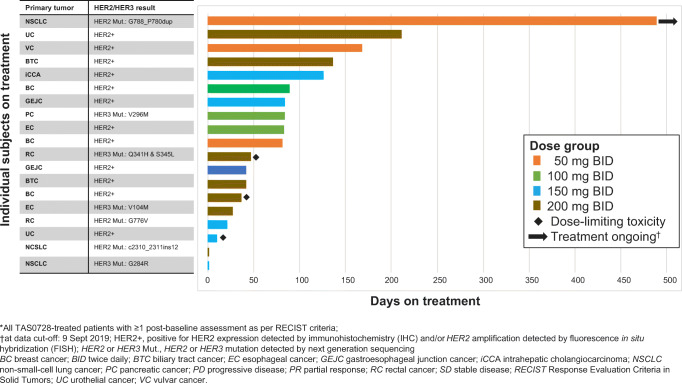
Duration of exposure (all treated population)*

Between 22 March 2018 and 29 March 2019, 25 patients were enrolled from six centers in the USA, France, Spain, the UK, and South Korea. Of these, four patients failed screening prior to receiving study drug, two did not receive study drug for other reasons, and 19 patients were treated with the study drug (all treated population). Baseline characteristics of the patients who received TAS0728 treatment per the protocol for 21-day cycles are summarized in Table 1. Seven patients received study drug at the highest dose administered in this study (200 mg BID) before a dose reduction to 150 mg BID was conducted in 6 patients. For the overall all treated population, the median treatment duration was 81.0 days (range 1–489 days) and the median number of cycles was 4.6 (Fig. 1). The mean relative dose intensity (actual amount of dose administered/amount of planned dose, %) was 74.9% (range 5%–100%). Across the 19 patients who received ≥1 dose of TAS0728, the mean age was 57.7 years (range 29–79) and all had an ECOG performance status of 0 or 1. Breast cancer and non-small-cell lung cancer (NSCLC) (*n* = 3 patients each) were the most common sites of primary tumors among the patients in the study. The mean time since initial cancer diagnoses was 31.5 months. All 19 treated patients had received ≥2 prior lines of systemic therapy. *HER2* amplification was detected in 7 (36.8%) of the 19 treated patients, and 6 of the patients (31.6%) had *HER2* overexpression at baseline. Three (15.7%) and four (21.0%) patients had *HER2* or *HER3* mutations, respectively (Table 1). One patient in the 150 mg BID cohort had *HER2* amplification and a *HER2* mutation (G776V).

**Table 1:** Patient baseline demographics and disease characteristics (all treated population)

	**Dose level of TAS0728**				
**Characteristic**	**50 mg BID** **(*N*** **= 3)**	**100 mg BID** **(*N*** **= 3)**	**150 mg BID** **(*N*** **= 6)**	**200 mg BID** **(*N*** **= 7)**	**Overall** **(*N*** **= 19)**
**Age (years)**					
Mean (SD)	67.3 (13.3)	62.0 (7.0)	52.2 (14.2)	56.6 (13.4)	57.7 (13.1)
Range	52, 76	57, 70	29, 66	38, 79	29, 79
**Sex, n (%)**					
Male	1 (33.3)	2 (66.7)	4 (66.7)	3 (42.9)	10 (52.6)
Female	2 (66.7)	1 (33.3)	2 (33.3)	4 (57.1)	9 (47.4)
**ECOG PS, n (%)**					
0	1 (33.3)	1 (33.3)	1 (16.7)	4 (57.1)	7 (36.8)
1	2 (66.7)	2 (66.7)	5 (83.3)	3 (42.9)	12 (63.2)
**Weight (kg)**					
Mean (SD)	71.6 (3.1)	62.4 (8.5)	75.2 (12.8)	58.9 (8.6)	66.6 (11.6)
Range	68, 74	53,69	61, 92	46, 71	46, 92
**Site of primary tumor, n (%)**					
Biliary tract cancer	0	0	1 (16.7)	2 (28.6)	3 (15.8)
Breast cancer	1 (33.3)	1 (33.3)	0	1 (14.3)	3 (15.8)
Esophagus cancer	0	1 (33.3)	0	1 (14.3)	2 (10.5)
Gastric and GEJ cancer	0	0	2 (33.3)	0	2 (10.5)
Malignant neoplasm of the vulva	1 (33.3)	0	0	0	1 (5.3)
NSCLC	1 (33.3)	0	1 (16.7)	1 (14.3)	3 (15.8)
Pancreas cancer	0	1 (33.3)	0	0	1 (5.3)
Rectum cancer	0	0	1 (16.7)	1 (14.3)	2 (10.5)
Urothelial cancer	0	0	1 (16.7)	1 (14.3)	2 (10.5)
**HER2 overexpression**	1 (33.3)	1 (33.3)	3 (50.0)	1 (14.3)	6 (31.6)
HER2 IHC 3+	1 (33.3)	1 (33.3)	3 (50.0)	1 (14.3)	6 (31.6)
***HER2*** **amplification**	1 (33.3)	1 (33.3)	2 (33.3)^a^	3 (42.9)	7 (36.8)^a^
***HER2*** **mutation**	1 (33.3)	0	1 (16.7)^a^	1 (14.3)	3 (15.8)^a^
***HER3*** **mutation**	0	1 (33.3)	1 (16.7)	2 (28.6)	4 (21.1)

*BID* twice daily, *ECOG PS* Eastern Cooperative Oncology Group performance status, *GEJ* Gastroesophageal junction, *NSCLC* Non-small-cell lung cancer, *SD* Standard deviation

^a^ One patient in the 150 mg BID cohort had *HER2* amplification and a *HER2* mutation (G776V)

Of the 19 patients treated with TAS0728, 18 (94.7%) discontinued treatment, most (*n* = 14, 73.7%) due to disease progression. Reasons for treatment discontinuation in the other patients were fatal cardiac arrest (1 patient; 5.3%), AEs (1 patient; 5.3%); and patient decision to withdraw from treatment (*n* = 2; 10.5%). At the time of data cut-off for the final analysis (9 September 2019), one patient was still receiving TAS0728 (Fig. 1); this patient had received TAS0728 50 mg BID for 489 days at that date and at the time of writing (October 2020) was continuing to receive TAS0728 treatment.

#### Safety

The study was stopped due to unacceptable toxicity during the dose-escalation, therefore the MTD was not determined. Overall, the incidence of AEs reported during the study was higher at doses of 150 mg and 200 mg BID TAS0728 compared with lower doses (Table 2); however, all 19 (100.0%) patients in the all treated population experienced an AE during the study at all doses administered, most of which were considered by the investigator to be treatment-related (Table 3).

**Table 2:** Overview of treatment-emergent adverse events by TAS0728 dose level (all treated population)

	**Dose level of TAS0728**				
	**50 mg BID** **(*N*** **= 3)** **n, (%)**	**100 mg BID** **(*N*** **= 3)** **n, (%)**	**150 mg BID** **(*N*** **= 6)** **n, (%)**	**200 mg BID** **(*N*** **= 7)** **n, (%)**	**Overall** **(*N*** **= 19)** **n, (%)**
Patients with AEs	3 (100)	3 (100)	6 (100)	7 (100)	19 (100)
Patients with SAEs	1 (33.3)	0	4 (66.7)	3 (42.9)	8 (42.1)
Patients with DLT AEs	0	0	1 (16.7)	2 (28.6)	3 (15.8)
Patients with Grade ≥ 3 AEs	2 (66.7)	0	3 (50.0)	4 (57.1)	9 (47.4)
Patients with treatment-related AEs	3 (100)	3 (100)	5 (83.3)	6 (85.7)	17 (89.5)
Patients with treatment-related and Grade ≥ 3 AEs	0	0	2 (33.3)	4 (57.1)	6 (31.6)
Patients with AEs that led to study treatment discontinuation	0	1 (33.3)	1 (16.7)	1 (14.3)	3 (15.8)
Patients with AEs that had an outcome of death	0	0	1 (16.7)	0	1 (5.3)

*AE* Adverse event, *BID* Twice daily, *DLT* Dose-limiting toxicity, *SAE* Serious adverse event

**Table 3:** Treatment-related adverse events experienced in ≥10% Patients by dose level and severity (all treated population^a^)

	**Grade 1 n (%)**	**Grade 2 n (%)**	**Grade 3 n (%)**	**Grade 4 n (%)**	**Grade 5 n (%)**	**Total n (%)**	**≥Grade 3** **n (%)**
	**Dose Level 1: 50 mg BID (*N*** **= 3)**						
Adverse events experienced in ≥10% patients	3 (100)	1 (33.3)	2 (66.7)	1 (33.3)	0	3 (100)	2 (66.7)
Diarrhea	2 (66.7)	1 (33.3)	1 (33.3)	0	0	2 (66.7)	1 (33.3)
Hyperuricemia	2 (66.7)	0	0	0	0	2 (66.7)	0
	**Dose Level 2: 100 mg BID (*N*** **= 3)**						
Adverse events experienced in ≥10% patients	3 (100)	2 (66.7)	0	0	0	3 (100)	0
Diarrhea	3 (100)	1 (33.3)	0	0	0	3 (100)	0
	**Dose Level 3d: 150 mg BID (*N*** **= 6)**						
Adverse events experienced in ≥10% patients	5 (83.3)	5 (83.3)	3 (50.0)	0	1 (16.7)	6 (100)	3 (50.0)
Diarrhea	2 (33.3)	1 (16.7)	2 (33.3)	0	0	4 (66.7)	2 (33.3)
Vomiting	2 (33.3)	0	0	0	0	2 (33.3)	0
Pyrexia	0	2 (33.3)	0	0	0	2 (33.3)	0
Back pain	1 (16.7)	1 (16.7)	0	0	0	2 (33.3)	0
	**Dose Level 3: 200 mg BID (*N*** **= 7)**						
Adverse events experienced in ≥10% patients	6 (85.7)	7 (100)	4 (57.1)	0	0	7 (100)	4 (57.1)
Diarrhea	5 (71.4)	5 (71.4)	3 (42.9)	0	0	6 (85.7)	3 (42.9)
Anemia	2 (28.6)	3 (42.9)	1 (14.3)	0	0	5 (71.4)	1 (14.3)
Cough	3 (42.9)	0	0	0	0	3 (42.9)	0
Dermatitis acneiform	2 (28.6)	1 (14.3)	0	0	0	3 (42.9)	0
Fatigue	1 (14.3)	3 (42.9)	0	0	0	3 (42.9)	0
Pyrexia	2 (28.6)	1 (14.3)	0	0	0	3 (42.9)	0
Nausea	1 (14.3)	2 (28.6)	0	0	0	2 (28.6)	0
Vomiting	1 (14.3)	1 (14.3)	0	0	0	2 (28.6)	0
Asthenia	2 (28.6)	1 (14.3)	0	0	0	2 (28.6)	0
Oedema peripheral	2 (28.6)	0	0	0	0	2 (28.6)	0
Dry skin	2 (28.6)	0	0	0	0	2 (28.6)	0
Urinary tract infection	0	2 (28.6)	0	0	0	2 (28.6)	0
Decreased appetite	1 (14.3)	1 (14.3)	0	0	0	2 (28.6)	0
Hypokalemia	2 (28.6)	0	0	0	0	2 (28.6)	0

*TRAE* Treatment-related adverse event

^a^Table includes TRAEs occurring in ≥10% of TAS0728 treated patients at any grade between first dose and 30 days after last dose of study drug

The TRAEs with the highest incidence (≥20% of patients) were diarrhea (78.9%), nausea (21.1%), vomiting (21.1%), and fatigue (21.1%). Almost one third (31.6%) of the TRAEs experienced overall were considered ≥Grade 3 in severity, with diarrhea having the highest incidence (26.3%). Other ≥Grade 3 TRAEs were acute kidney injury, proteinuria, and cardiac arrest (1 patient, 5.3% each). When broken down by dose level, the highest incidence of AEs experienced at each dose level cohort was diarrhea. At the 50-mg dose level cohort, 2 of 3 (66.7%) patients had diarrhea, 1 at a severity ≥Grade 3. Hyperuricemia was also experienced in 2 of 3 (66.7%) patients at this dose. At the 100-mg dose level cohort, all 3 (100%) patients experienced diarrhea, all at a severity of Grade 1 or Grade 2. At the 200-mg dose, 6 of 7 (85.7%) patients experienced diarrhea, 3 at a severity ≥Grade 3. Other AEs experienced at this dose in ≥2 patients were anemia (5/7 patients, 71.4%), cough, dermatitis acneiform, fatigue, and pyrexia (3/7 patients each, 42.9%), and nausea, vomiting, asthenia, edema peripheral, dry skin, urinary tract infection, decreased appetite, and hypokalemia (2/7 patients each, 28.6%). At the reduced dose of 150 mg BID, 4 of 6 (66.7%) patients experienced diarrhea, 2 at a severity ≥Grade 3. Other AEs experienced at this dose in ≥2 patients were vomiting, pyrexia, and back pain.

Two patients at the 200-mg BID dose level experienced a DLT of diarrhea ≥Grade 3 that lasted >48 h and was unresponsive to intensive antidiarrheal medication (Table 4). Subsequently, the dose of TAS0728 was reduced to 150 mg BID. At this dose, 1 patient experienced a DLT of diarrhea ≥Grade 3 that lasted >48 h and was unresponsive to intensive antidiarrheal medication.

**Table 4:** Dose limiting toxicities on TAS0728 treatment (DLT-evaluable population)

	**50 mg BID** **(*N*** **= 3)** **n, (%)**	**100 mg BID** **(*N*** **= 3)** **n, (%)**	**150 mg BID** **(*N*** **= 3)** **n, (%)**	**200 mg BID** **(*N*** **= 6)** **n, (%)**	**Total** **(*N*** **= 15)** **n, (%)**
Any DLTs	0	0	1 (33.3)	2 (33.3)	3 (20.0)
Grade ≥ 3 diarrhea only if lasting >48 h and unresponsive to intensive antidiarrheal medication	0	0	1 (33.3)	2 (33.3)	3 (20.0)

*BID* Twice daily, *DLT* Dose-limiting toxicity

A total of 4 patients died during the study; 3 of 4 deaths occurred >30 days after the last dose of study treatment, all due to clinical progression. The other patient had an SAE with an outcome of death: this patient had a cardiac arrest after completing 1 cycle (21 days) of TAS0728 at a dose of 150 mg BID. This patient was a 42-year-old male patient with colorectal cancer and metastases to the liver and lungs, with no known history of heart disease. No abnormal results had been observed in his screening or in-study ECG assessments. The cardiac arrest occurred during the patient’s second cycle of TAS0728. After onset of dizziness and loss of consciousness, the patient underwent defibrillation by emergency services before being taken to hospital. Thereafter, the patient was unresponsive, intubated, and started on amiodarone drip for arrhythmia. Diagnostic workup was negative for coronary ischemia (troponin I was not elevated). Blood cultures were negative, and no acute cardiopulmonary abnormality was seen on chest x-ray. CT of the head showed loss of normal gray-white matter differentiation with decreased sulcation over the cerebral hemispheres bilaterally suggesting global anoxic brain ischemia. The patient died the following morning. The investigator and sponsor considered the event of cardiac arrest possibly related to TAS0728.

In the overall population, 8 (42.1%) of 19 patients experienced ≥1 SAE. Of these, five (26.3%) patients experienced an SAE that was Grade 3 or higher in severity. Serious AEs were experienced at dose level cohorts of 50 mg BID, 200 mg BID, and 150 mg BID, and included pyrexia (4 patients, 21.1%), diarrhea (2 patients, 10.5%), and dysphagia, cellulitis, clostridium colitis, cardiac arrest, back pain, and acute kidney injury (1 patient each, 5.3%). The SAE with the overall highest incidence was pyrexia, which occurred in 4 patients (2 each at doses of 200 mg and 150 mg BID). Three (15.8%) of the 19 patients in the all treated population experienced an SAE considered related to study drug, including SAEs of diarrhea (200- and 150-mg BID) and cardiac arrest (150 mg BID).

In total, 4 patients experienced an AE that led to study drug discontinuation. At the dose level of TAS0728 100 mg BID one patient experienced blood albumin decreased (Grade 2), one patient experienced myalgia (Grade 1) and abdominal pain (Grade 1). At the dose level of TAS0728 150 mg BID, one patient experienced cardiac arrest, (Grade 5/fatal). At the dose level of TAS0728 200 mg BID, one patient experienced acute kidney injury (Grade 3) and weight decreased (Grade 2).

No clinically meaningful changes from baseline were noted during the study for clinical laboratory results or for standard 12-lead ECG parameters. All patients had an ECOG performance status score of 0 or 1 at baseline. In all patients having an ECOG score of 0 at baseline, this score worsened to 1 during the study. Most of the patients with an ECOG score of 1 at baseline did not have a change in status during the study; however, 2 patients (1 in each of the 200-mg and 150-mg BID cohorts) worsened to an ECOG status of 2 during the study.

#### Pharmacokinetics

PK parameters of TAS0728 on cycle 1, day 1 and cycle 2, day 1 are summarized in Table 5, respectively. Absorbed TAS0728 reached C_max_ at approximately 0.5 to 4 h after oral administration, and then declined with t_1/2_ of approximately 2.0 h on cycle 1, day 1. Covariability values of C_max_ and AUCs on cycle 1, day 1 were from 16.2% to 97.8%. Due to the dose interruption/reduction or discontinuation, the number of patients who represented accurately the steady-state PK on cycle 2, day 1 was limited. T_max_ and t_1/2_ values observed at the start of cycle 2 were similar to those on Cycle 1, Day 1. No significant accumulation of TAS0728 exposure was observed following the BID multiple-dose administration.

**Table 5:** Pharmacokinetic parameters of TAS0728 on cycle 1, day 1

**Visit**		**Cycle 1, day 1**						
**Planned dose (mg)**	**Statistic**	**C**_**max**_ **(ng/mL)**	**T**_**max**_ **(hr)**^**a**^	**AUC**_**last**_ (hr*ng/mL)	**AUC**_**0–12**_ (hr*ng/mL)		**t1/2** **(hr)**	**AUC**_**inf**_ (hr*ng/mL)
**50**	**n**	3	3	3	3		3	3
	**Mean**	1162	0.50	2415	2437		1.96	2470
	**SD**	347	0.50	423	394		0.27	415
	**CV%**	29.8	1.58	17.5	16.2		13.7	16.8
**100**	**n**	3	3	3	3		3	3
	**Mean**	2039	0.53	5626	5669		1.90	5765
	**SD**	1995	0.58	5226	5277		0.16	5352
	**CV%**	97.8	3.05	92.9	93.1		8.6	92.8
**200**	**n**	7	7	7	6		6	6
	**Mean**	5091	0.45	15,108	14,653		2.05	15,046
	**SD**	2168	1.00	6740	7198		0.33	7596
	**CV%**	42.6	3.92	44.6	49.1		16.4	50.5
**150**	**n**	6	6	6	5		5	5
	**Mean**	5402	0.50	19,523	12,946		1.91	13,158
	**SD**	2412	0.59	16,832	5281		0.21	5447
	**CV%**	44.6	1.05	86.2	40.8		11.0	41.4
**Visit**		**Cycle 2, day 1**						
**Planned dose (mg)**	**Statistic**	**C**_**max**_ **(ng/mL)**	**T**_**max**_ **(hr)**^**a**^	**AUC**_**last**_ (hr*ng/mL)	**AUC**_**0–12**_ (hr*ng/mL)	**t**_**1/2**_ **(hr)**	**R (C**_**max**_**)**	**R(AUC**_**0–12**_**)**
**50**	**n**	0	0	0	0	0	0	0
	**Mean**	NA	NA	NA	NA	NA	NA	NA
	**SD**	NA	NA	NA	NA	NA	NA	NA
	**CV%**	NA	NA	NA	NA	NA	NA	NA
**100**	**n**	3	3	3	2	2	3	2
	**Mean**	1389	0.92	4276	5475	2.17	0.56	0.87
	**SD**	1765	3.03	3860	NA	NA	0.29	NA
	**CV%**	127.1	6.20	90.3	NA	NA	52.2	NA
**200**	**n**	2	2	2	2	2	2	2
	**Mean**	2565	0.52	11,248	11,459	3.13	0.96	1.18
	**SD**	NA	1.88	NA	NA	NA	NA	NA
	**CV%**	NA	3.25	NA	NA	NA	NA	NA
**150**	**n**	2	2	2	2	2	2	2
	**Mean**	3460	1.02	14,782	14,782	3.11	0.91	1.61
	**SD**	NA	1.30	NA	NA	NA	NA	NA
	**CV%**	NA	1.58	NA	NA	NA	NA	NA

*AUC*_*0–12*_ Area under the plasma concentration-time curve from the time 0 to the time 12 h, *AUC*_*inf*_ area under the plasma concentration-time curve from 0 time to infinity, AUC_last_ area under the plasma concentration-time curve from the time 0 to the time of the last plasma concentration, *C*_*max*_ maximum observed plasma concentration, *CV* Coefficient of variation, *N* Number of observation, *NA* Not applicable, *R(AUC*_*0–12*_*)* Observed accumulation ratio of AUC_0–12_, *R(C*_*max*_*)* Observed accumulation ratio of C_max_, *SD* Standard deviation, *T*_*1/2*_ Terminal elimination half-life, *T*_*max*_ Time to reach maximum observed plasma concentration

^**a**^For T_max_, the values shown represent minimum, median, and maximum

#### Clinical activity

**Fig. 2:**
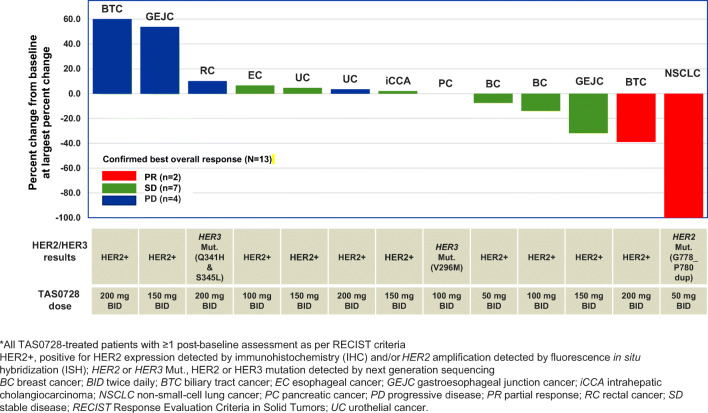
Waterfall plot of best change from baseline in the size of target lesions for patients with tumor response data*

Out of a total of 19 treated patients, a total of two objective responses were observed: a partial response (PR) in a patient with NSCLC and *HER2* mutation (G788_P780dup) treated at 50 mg BID and a PR in a patient with biliary tract cancer (BTC) with *HER2* amplification confirmed by FISH treated at 200 mg BID. In all, disease control (best overall tumor response of PR or stable disease [SD]) was observed in 10 patients (Fig. 2). Note that 1 patient in the 50-mg BID group had only non-target lesions (therefore there was no change in target lesion from baseline), this patient was included in the 14 patients with tumor response data (as a best response of SD), but not included in Fig. 2.

### Discussion

This phase I study was intended to assess the safety and tolerability of TAS0728 in patients with advanced solid tumors harboring *HER2* or *HER3* aberrations. Following escalation of the dose to 200 mg BID, two DLTs were observed, both cases of Grade 3 diarrhea lasting >48 h and not responsive to intensive antidiarrheal treatment. Following de-escalation of the dose to 150 mg BID, another DLT of Grade 3 diarrhea lasting >48 h and not responsive to intensive antidiarrheal treatment was observed in one patient.

In nonclinical studies, the inhibitory effect of TAS0728 was much greater against *HER2* and *HER3* than against EGFR, and administration of TAS0728 did not induce diarrhea during the treatment at efficacious doses in mouse models [2]; accordingly, it was hypothesized that treatment with TAS0728 might result in a lower incidence of AEs characteristically associated with EGFR inhibition, including gastrointestinal and cutaneous toxicity. However, in this study the majority of patients experienced one or more of these toxicities. At the 150 mg and 200 mg BID doses, these toxicities were significant. Moreover, a fatal instance of cardiac arrest occurred in a patient with no prior history of heart disease and in whom other reasons related to cardiac arrest were not identified. Cardiac arrest as a secondary event is not typical of the recognized cardiotoxicity observed during treatment with *HER2*-targeted therapies, which manifests as decreased left ventricular ejection fraction and/or symptomatic heart failure dysfunction [9]. The etiology of the cardiac arrest event within the current study was unclear, however causal relationship to TAS0728 could not be excluded due to the temporal association observed.

Although evaluation of efficacy was not a primary objective of the dose escalation portion of this study, some evidence of clinical benefit was obtained; this included two PRs among 14 patients evaluable for best overall response. However, considering the toxicity profile observed in the study, and taking into account the fatal AE of cardiac arrest considered possibly related to TAS0728, the sponsor determined that the overall risk-benefit ratio no longer favored the dose level tested in this study.

Of the two patients with PRs to TAS0728, one patient had BTC with *HER2* amplification (FISH-confirmed). This patient completed 136 days on TAS0728 treatment at 200 mg BID before disease progression and had not required dose adjustment. In contrast, in two patients with breast cancer and esophageal cancer, who had FISH-confirmed *HER2* amplification, disease progression occurred within around 80 days of starting TAS0728 treatment at 50 mg BID and 100 mg BID, respectively. Neither of these patients had required dose adjustment. Overall, these data suggest that the PR in the patient with BTC may have been influenced by other factors additional to *HER2* amplification, which may also have enabled this patient to tolerate treatment with TAS0728 for longer than the time observed in the patients with *HER2* amplification whose disease progressed more rapidly despite treatment with TAS0728 at the same or higher BID dose level.

The other patient who achieved a PR on TAS0728 had NSCLC. This patient’s tumor carried a mutation in exon 20 of the *HER2* gene, which is a recognized mutation hotspot in the intracellular tyrosine kinase domain. Patients with tumors harboring this specific in-frame insertion, G778_P780dup, have been shown to respond to treatment with the irreversible pan-HER tyrosine kinase inhibitor neratinib [10]. This mutation results in insertion of a duplicated sequence of three amino acids, glycine-serine-proline, into the *HER2* protein. TAS0728 covalently binds to *HER2* near the site of this mutation hotspot at C805 and selectively inhibits its kinase activity [2]. At the time of termination of the present study, the patient with NSCLC who responded to TAS0728 treatment had received 50 mg BID for 489+ days and had not required dose adjustment while on the study drug. This suggests that in a patient whose tumor was responding to treatment, long-term tolerability at this dose level was acceptable.

In the present study, all patients were heavily pretreated and had a variety of tumor types. The small number of patients treated and the presence of already advanced disease at the time of enrolment limit the interpretation of efficacy, however the observation of two PRs and disease control in 10 patients should encourage further investigation of novel *HER2*-targeted approaches for solid tumors with *HER2* aberrations. Several approved oral small molecule *HER2*-inhibitory compounds such as lapatinib, afatinib, neratinib, and tucatinib are associated with severe diarrhea, requiring proactive management, including patient education combined with antidiarrheal medication and dose reductions/interruptions, particularly during the initial weeks of treatment [11, 12]. Thus, the occurrence of diarrhea as a DLT in the current study was consistent with the AE profile observed for other *HER2* inhibitors known to also have EGFR inhibitory effects. With appropriate management, this TRAE has been manageable for other members of the oral HER2 inhibitor class.
